# Synthesis, crystal structure and Hirshfeld surface analysis of 1-[3-(2-oxo-3-phenyl-1,2-di­hydro­quinoxalin-1-yl)prop­yl]-3-phenyl-1,2-di­hydro­quinoxalin-2-one

**DOI:** 10.1107/S2056989024004377

**Published:** 2024-05-17

**Authors:** Nadeem Abad, Joel T. Mague, Abdulsalam Alsubari, El Mokhtar Essassi, Abdullah Yahya Abdullah Alzahrani, Youssef Ramli

**Affiliations:** a Laboratory of Medicinal Chemistry, Drug Sciences Research Center, Faculty of Medicine and Pharmacy, Mohammed V University in Rabat, Morocco; b Laboratory of Heterocyclic Organic Chemistry Faculty of Sciences, Mohammed V University, Rabat, Morocco; c Department of Chemistry, Tulane University, New Orleans, LA, 70118, USA; d Laboratory of Medicinal Chemistry, Faculty of Clinical Pharmacy, 21 September University, Yemen; e Department of Chemistry, Faculty of Science and Arts, King Khalid University, Mohail Assir, Saudi Arabia; Katholieke Universiteit Leuven, Belgium

**Keywords:** crystal structure, hydrogen bond, di­hydro­quinoxaline, π-stacking, C—H⋯π(ring) inter­action

## Abstract

In the title compound, C_31_H_24_N_4_O_2_, the di­hydro­quinoxaline units are both essentially planar with the dihedral angle between their mean planes being 64.82 (4)°. The attached phenyl rings differ significantly in their rotational orientations with respect to the di­hydro­quinoxaline planes. In the crystal, one set of C—H⋯O hydrogen bonds form chains along the *b*-axis direction, which are connected in pairs by a second set of C—H⋯O hydrogen bonds. Two sets of π-stacking inter­actions and C—H⋯π(ring) inter­actions join the double chains into the final three-dimensional structure.

## Abstract 2

In the title compound, the di­hydro­quinoxaline units are both essentially planar and the dihedral angle between their mean planes is 64.82 (2)°. In the crystal, C—H⋯O hydrogen bonds form chains along the *b*-axis direction which are joined by π-stacking and C—H⋯π(ring) inter­actions into the full three-dimensional network structure.

## 1. Chemical context

**Figure 1:**
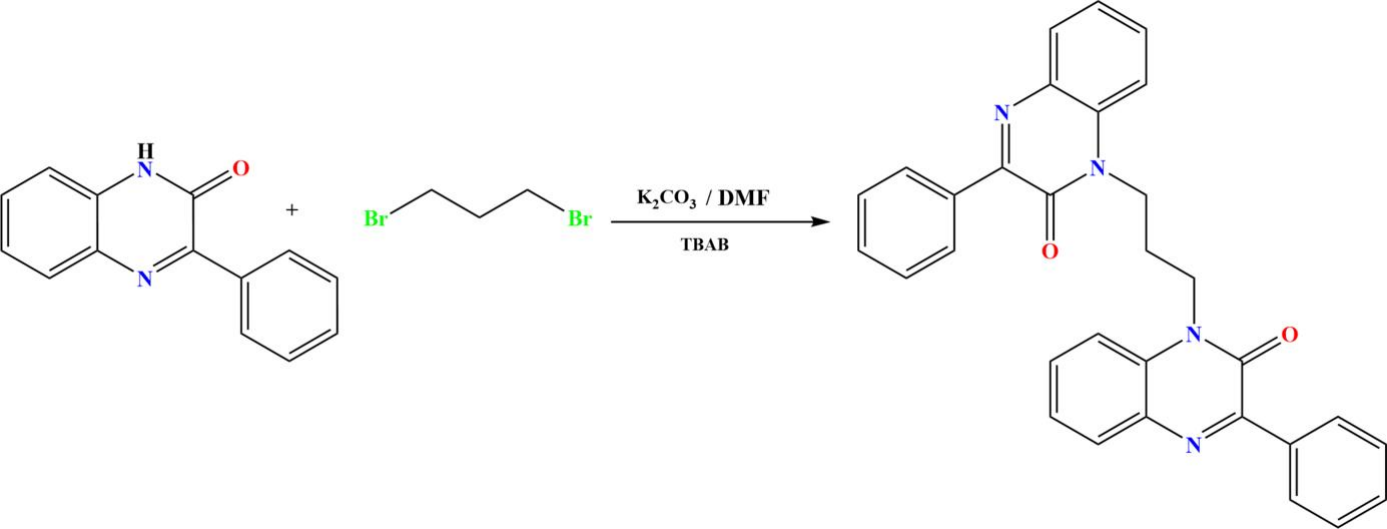
Synthesis of the title compound.

The family of nitro­genous drugs, notably those containing the quinoxaline moiety, is important in medicinal chemistry because of the wide range of pharmacological activities exhibited, including anti­bacterial, anti­tuberculosis, anti-inflammatory, anti­fungal anti-glycation, anti-analgesic and anti­cancer properties. In particular, quinoxalin-2-one derivatives are a class of heterocyclic compounds with different applications in various fields (Ramli *et al.*, 2014 ▸). They have been studied intensively as an important heterocyclic system for the synthesis of biologically active compounds ranging from herbicides and fungicides to therapeutically usable drugs (Ramli & Essassi, 2015 ▸). These chemicals are active anti-tumor agents with tyrosine kinase receptor inhibition properties (Galal *et al.*, 2014 ▸). They can also selectively antagonize the glycoprotein in cancer cells (Sun *et al.*, 2009 ▸). Quinoxalin-2-one derivatives are also potential antagonist ligands for imaging the A2A adenosine receptor by positron emission tomography (PET) (Holschbach et *al*., 2005 ▸). Given the wide range of therapeutic applications for such compounds, we have previously reported a route for the preparation of quinoxalin-2-one derivatives using *N*-alkyl­ation reactions carried out with di-halogenated carbon chains (Missioui *et al.* 2022 ▸; Abad *et al.*, 2024 ▸). A similar approach yielded the title compound, C_31_H_24_N_4_O_2_ (Fig. 1 ▸). In addition to the synthesis, we also report the mol­ecular and crystal structure along with a Hirshfeld surface analysis. 
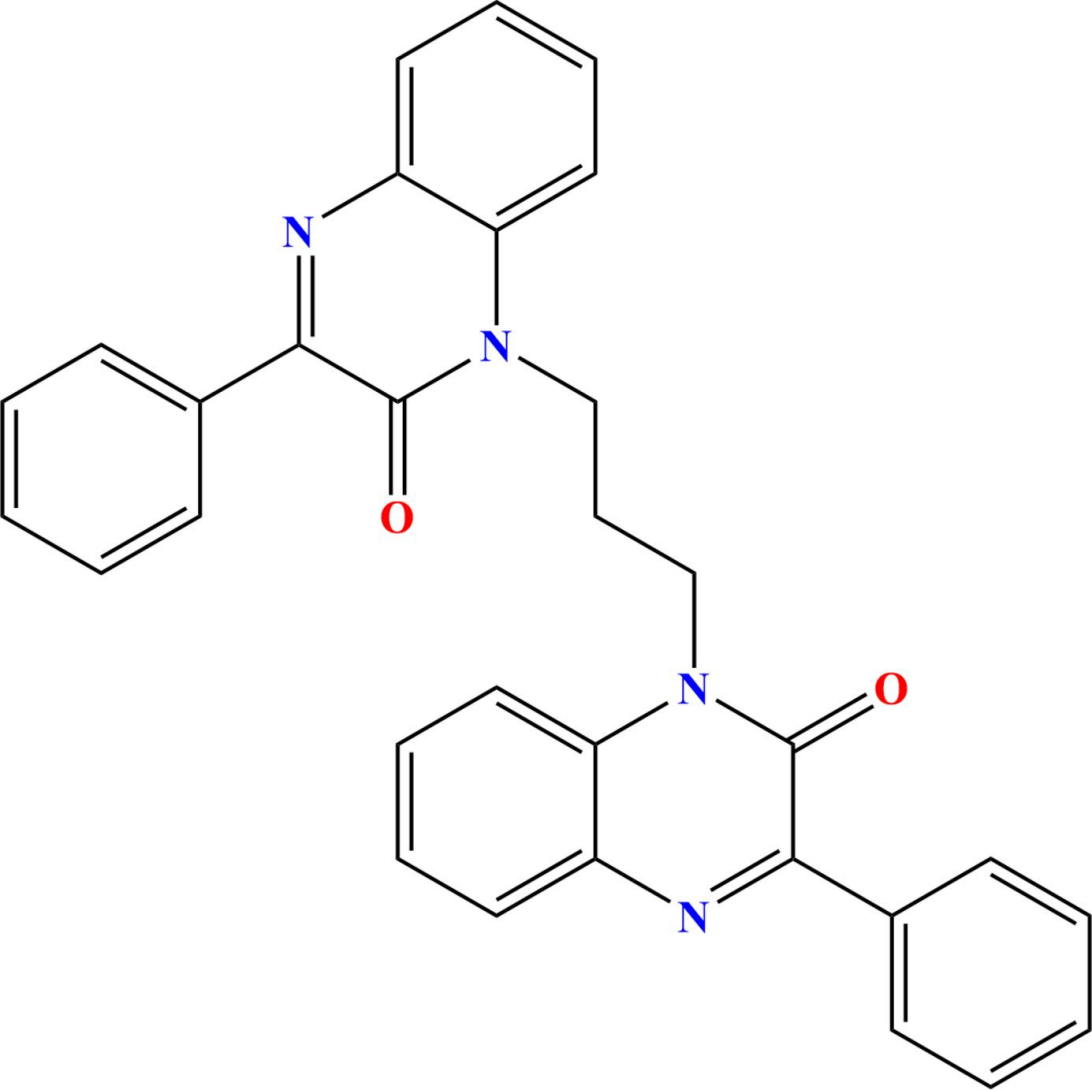


## 2. Structural commentary

**Figure 2:**
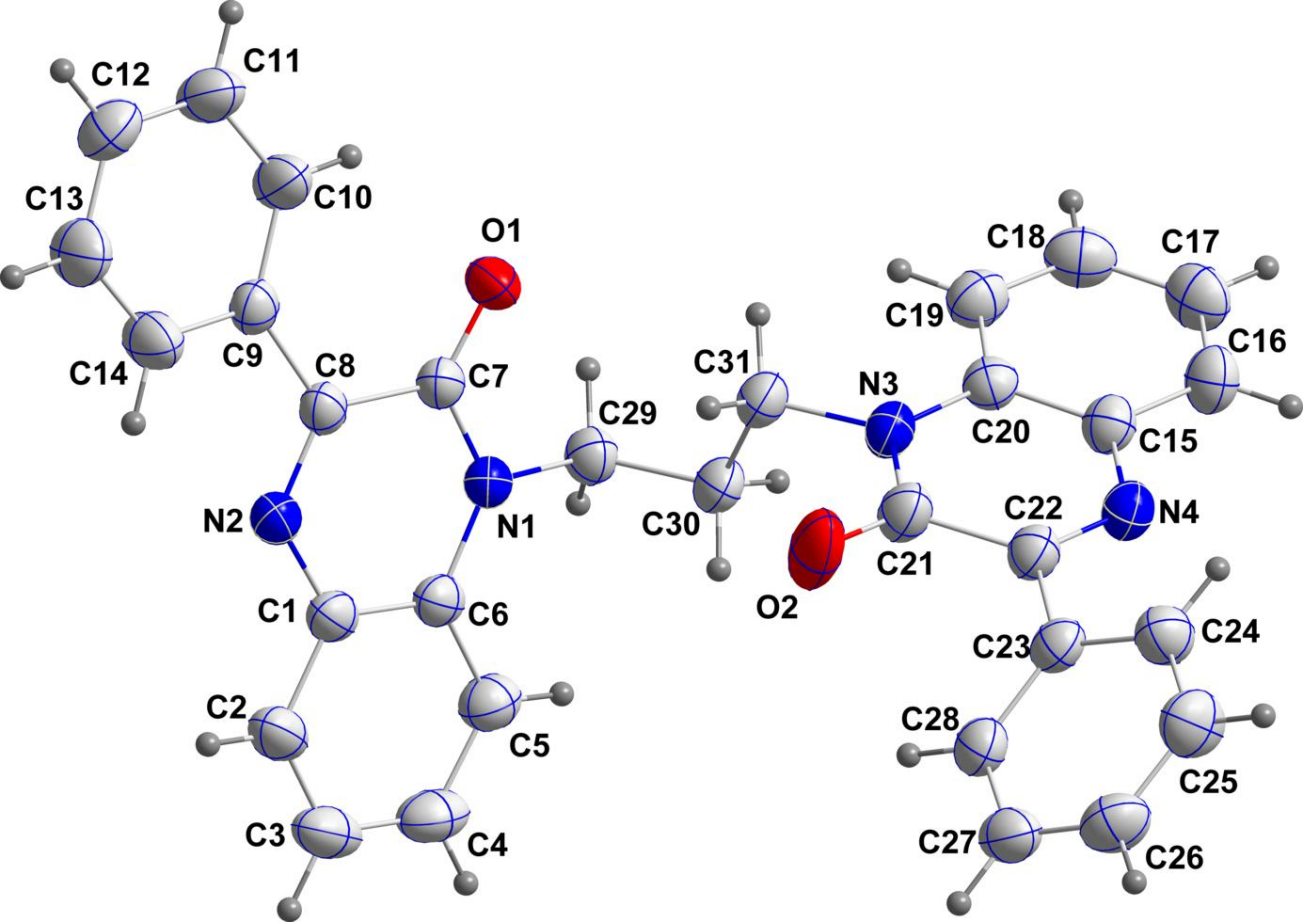
The title mol­ecule with labeling scheme and 50% probability ellipsoids.

**Figure 3:**
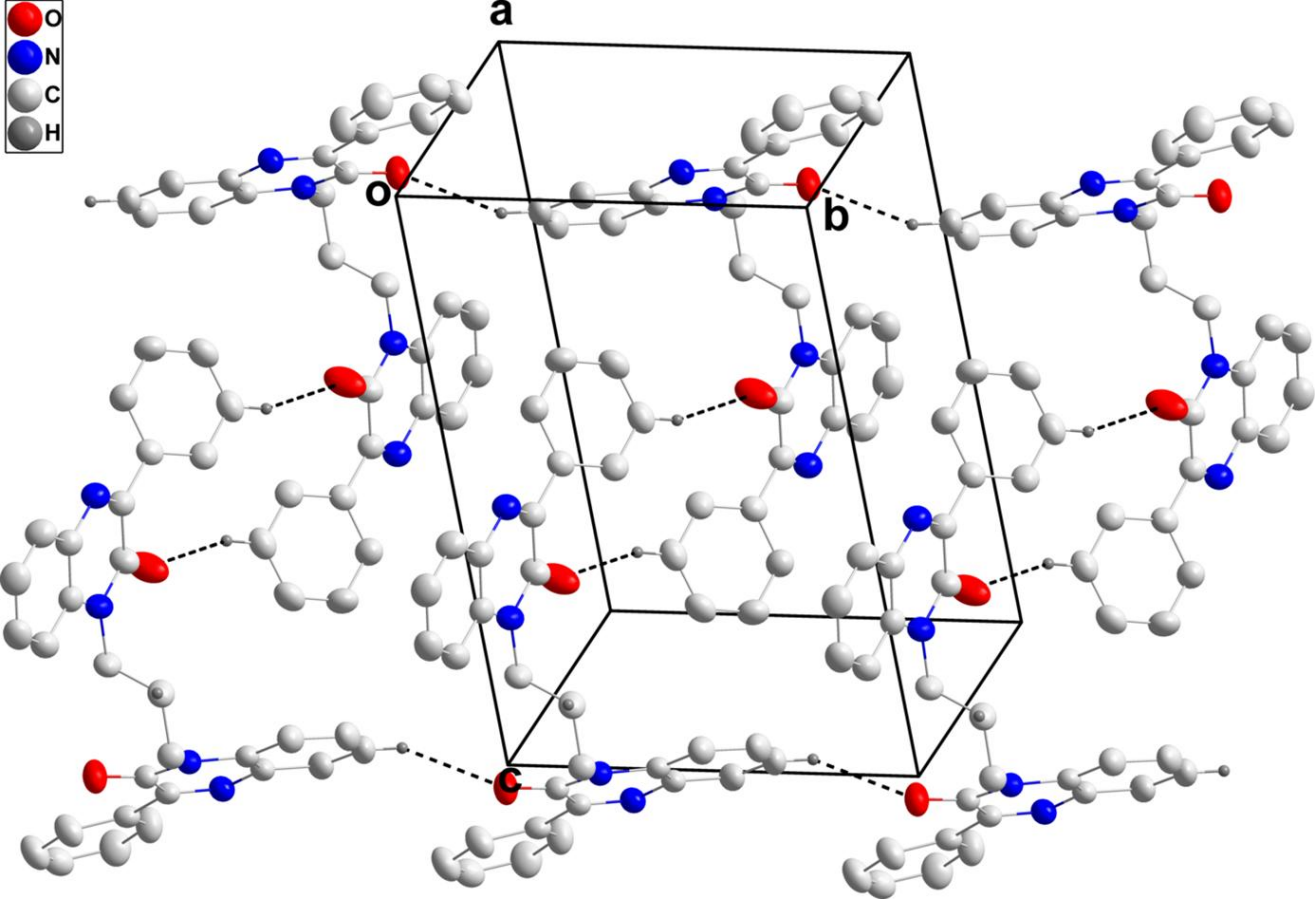
Perspective view of the chains formed by C—H⋯O hydrogen bonds (dashed lines).

The title compound crystallizes in the triclinic space group *P* 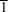 with one mol­ecule in the asymmetric unit (Fig. 2 ▸). The di­hydro­quinoxaline unit containing N1 is planar to within 0.038 (1) Å (r.m.s. deviation of fitted atoms = 0.0209 Å) while that containing N3 is planar to within 0.021 (1) Å (r.m.s. deviation = 0.0124 Å). The dihedral angle between their mean planes is 64.82 (4)°. The C9–C14 benzene ring is inclined to the plane of the di­hydro­quinoxaline unit containing N1 by 7.35 (5)°, which is due in part to an intra­molecular C10—H10⋯O1 hydrogen bond (Table 1 ▸). The corresponding angle on the other half of the mol­ecule is 37.63 (5)°. The greater out-of-plane orientation of the latter phenyl ring may be the result of its participation in C—H⋯π(ring) inter­actions (Table 1 ▸ and Fig. 3 ▸). There are close contacts of H29*A* with O1 (2.31 Å) and H31*B* with O2 (2.32 Å), which might be considered additional hydrogen-bond inter­actions although the C—H⋯O angles are only 102°. The central C—C—C unit extends out from N3 in an all-*trans* conformation with a C29—C30—C31—N3 torsion angle of −175.74 (9)° but this does not continue to the second quinoxaline unit as the N1—C29—C30—C31 torsion angle is −69.98 (13)°.

## 3. Supra­molecular features

**Figure 4:**
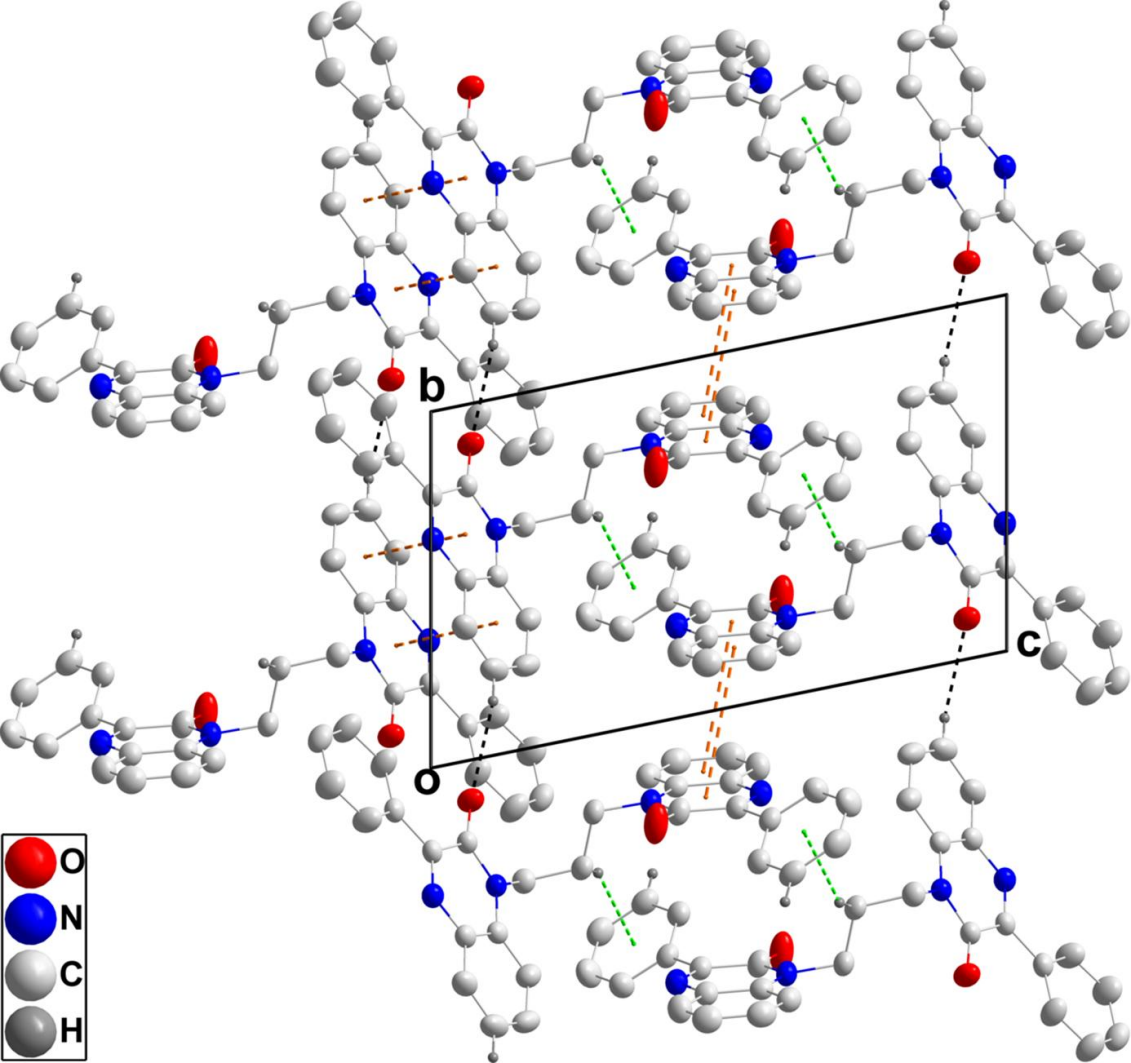
Packing viewed along the *a*-axis direction with C—H⋯O hydrogen bonds shown as black dashed lines and π-stacking and C—H⋯π(ring) inter­actions shown as orange and green dashed lines, respectively.

In the crystal, chains of mol­ecules extending along the *b*-axis direction are formed by C3—H3⋯O1 hydrogen bonds and are linked in pairs into a Z-shaped motif by C27—H27⋯O2 hydrogen bonds (Table 1 ▸ and Fig. 3 ▸). The paired chains are joined by π-stacking inter­actions between inversion-related di­hydro­quinoxaline moieties containing N1 (symmetry code: −*x* + 1, −*y* + 1, −*z*) with a distance of 3.5676 (7) Å between the centroids of the N1/C6/C1/N2/C8/C7 and C1–C6 rings as well as by corresponding inter­actions between those containing N3 (symmetry code: −*x* + 1, −*y* + 2, −*z* + 1) with a distance of 3.8641 (7) Å between the centroids of the N3/C20/C15/N4/C22/C21 and C15–C20 rings (Fig. 4 ▸). These inter­actions are accompanied by inversion-related C30—H30*A*⋯*Cg*6 inter­actions (Table 1 ▸ and Fig. 4 ▸; *Cg*6 is the centroid of ring C23–C28).

## 4. Database survey

**Figure 5:**
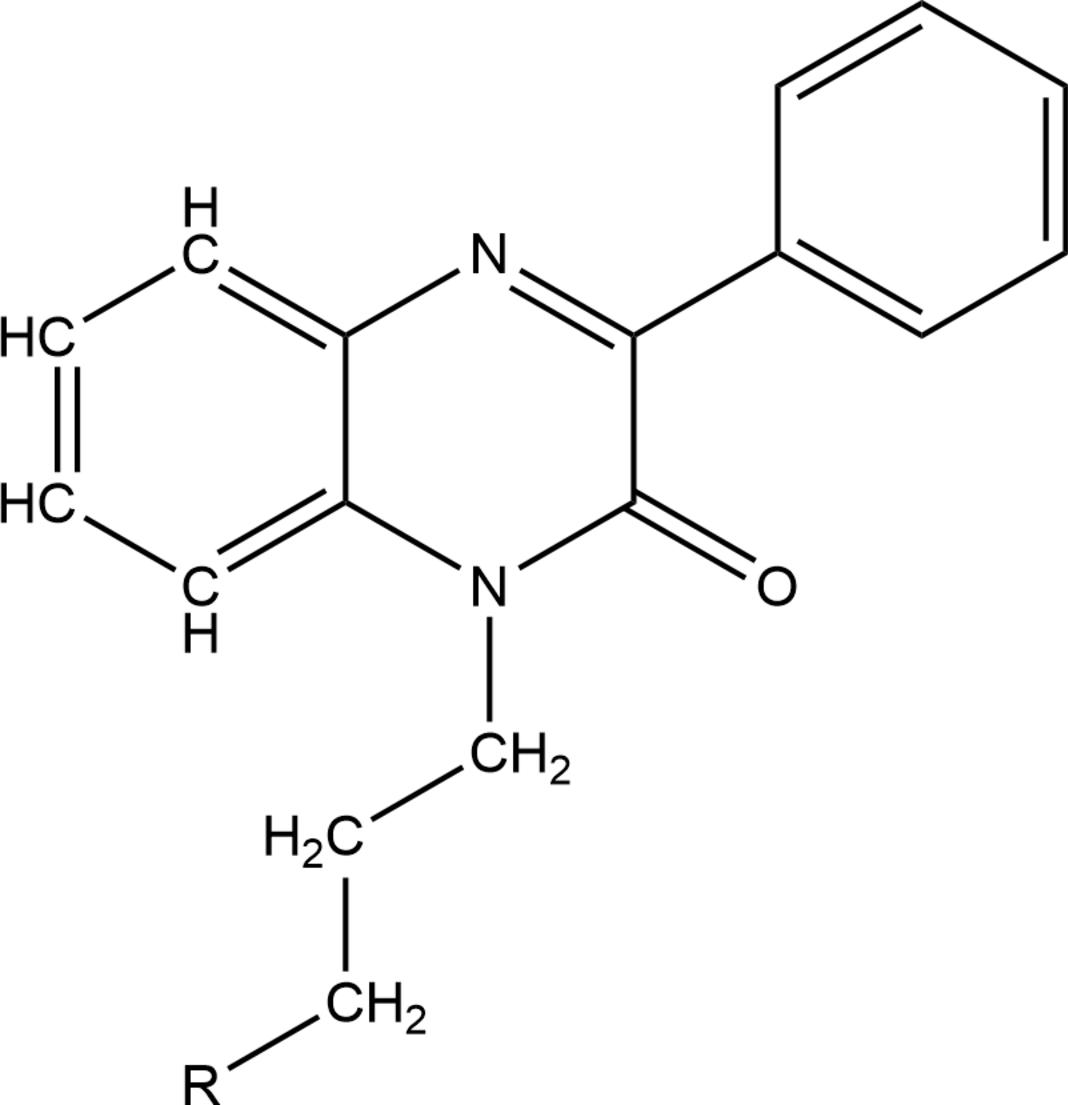
Search fragment used in the database survey.

A search of the Cambridge Structural Database (CSD, updated to March 2024; Groom *et al.*, 2016 ▸) with the search fragment shown in Fig. 5 ▸ (*R* = anything) yielded five hits. These contain *R* = *n*-pentyl (AZAZEC; Abad *et al.*, 2021*b* ▸), 2-oxy-3-phenyl­quinoxaline (KOPKAF; Abad *et al.*, 2024 ▸), OH (RIRBOM; Abad *et al.*, 2018 ▸), *n*-hexyl (UDAMIZ; Abad *et al.*, 2021*a* ▸) and Et (UFITEM; Abad *et al.*, 2023 ▸). In AZAZEC, the quinoxaline unit is planar with the exception of the nitro­gen bearing the alkyl chain while in the others, the unit shows somewhat greater deviations from planarity. The dihedral angle between the mean planes of the quinoxaline unit and the attached phenyl ring vary from 12.90 (4)° (AZAZEC) to 44.89 (3)° (RIRBOM) with the lower values resulting from intra­molecular C—H⋯O hydrogen bonding. In AZAZEC, RIRBOM and UFITEM there are C—H⋯π(ring) inter­actions, which help stabilize the crystal packing, while in UFITEM and KOPKAF there are π-stacking inter­actions between inversion-related quinoxaline moieties as in the present case. In UFITEM there are C=O⋯π(ring) inter­actions as well. In the examples containing a single quinoxaline moiety, the absolute values of the N—C—C—C torsion angles vary from 178.73 (8)° (KOPKAF) to 168.64 (8)° (RIRBOM) while in KOPKAF and RIRBOM, the O2—C17—C16—C15 torsion angles are, respectively, −68.46 (12) and −63.85 (11)°. These conformations are quite similar to that in the present structure.

## 5. Hirshfeld surface analysis

**Figure 6:**
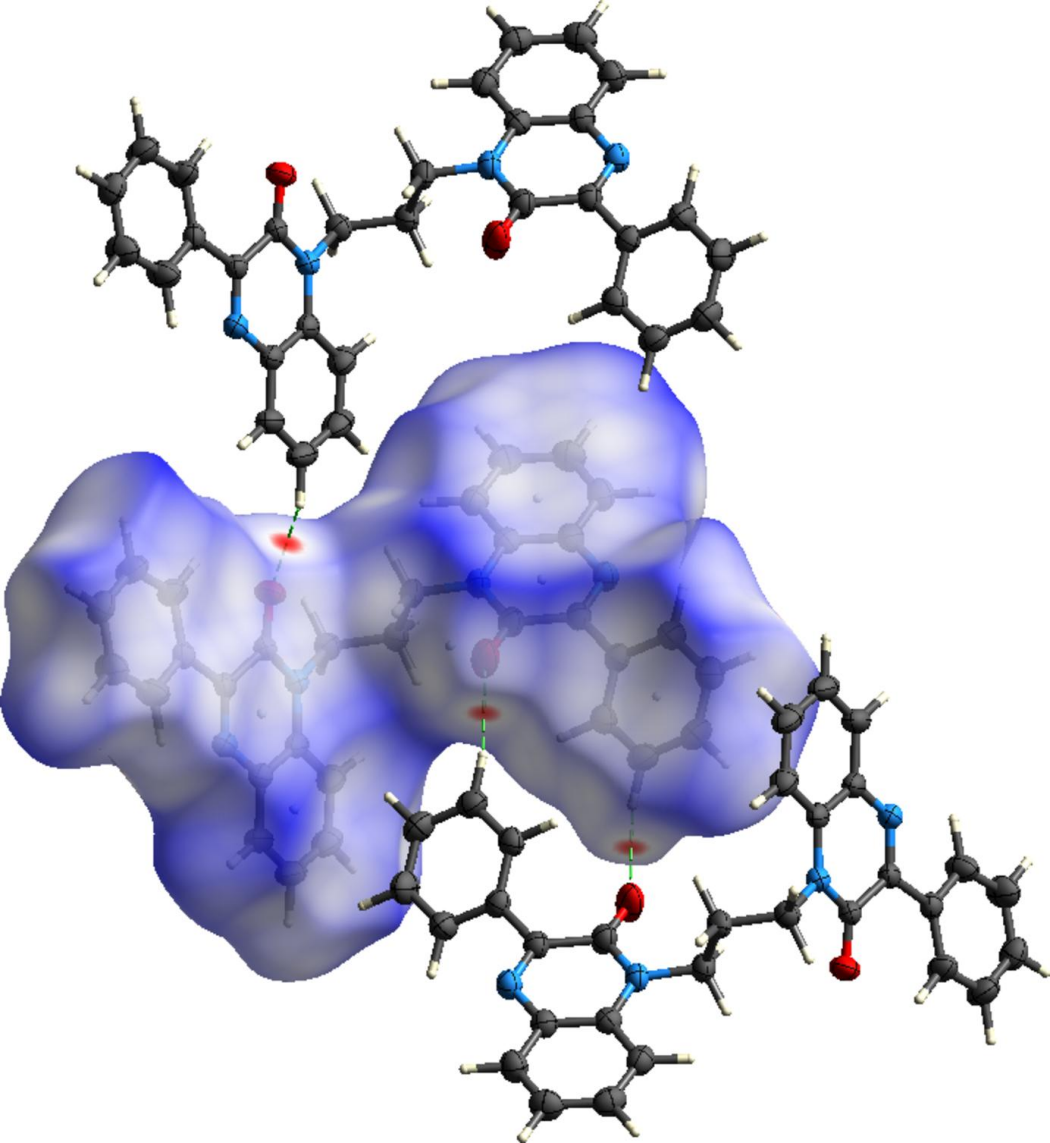
The Hirshfeld surface plotted over *d*_norm_ showing the C—H⋯O hydrogen bonds to neighboring mol­ecules.

**Figure 7:**
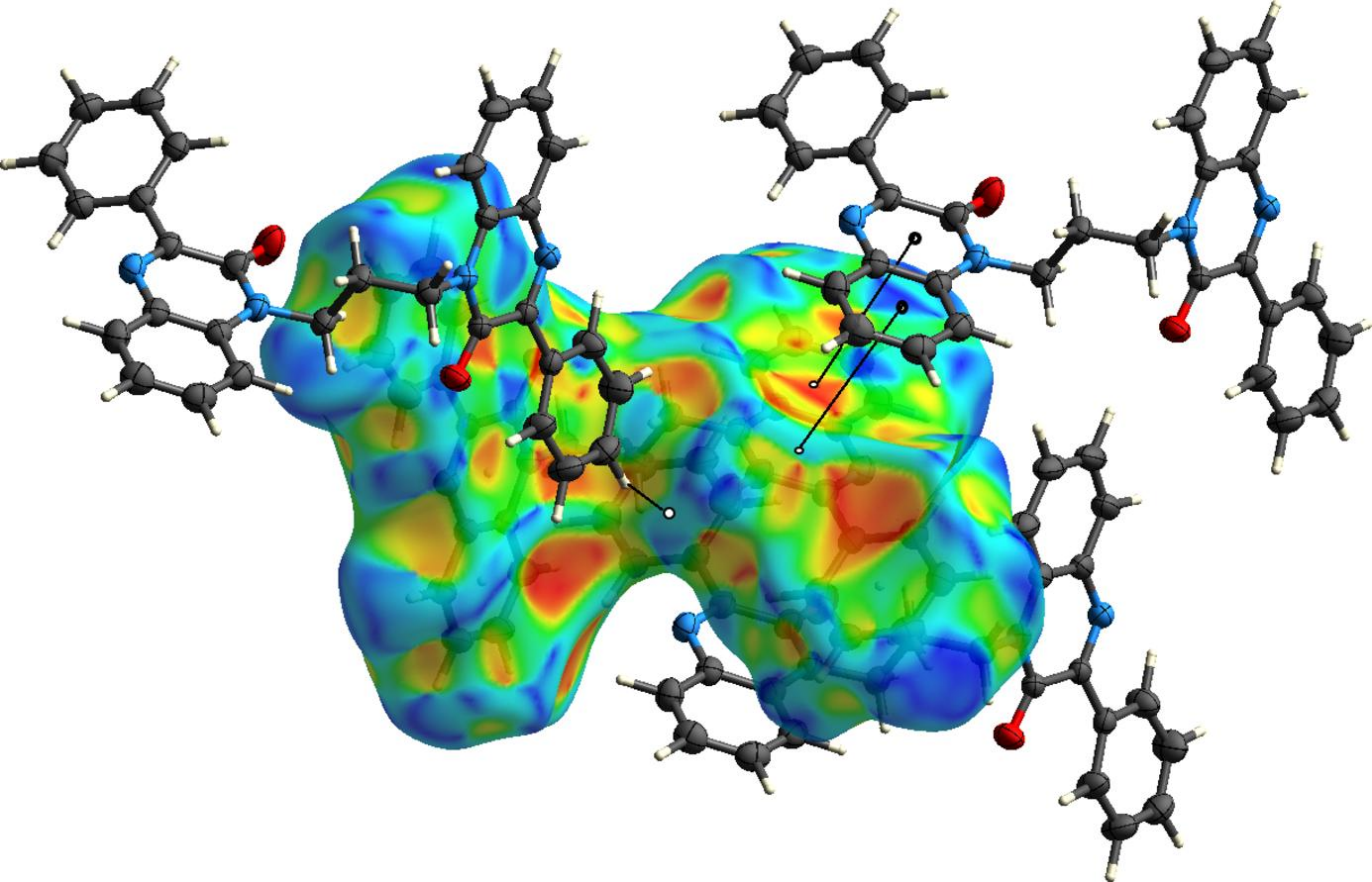
The Hirshfeld surface plotted over shape-index showing the π-stacking and C—H⋯π(ring) inter­actions to neighboring mol­ecules.

**Figure 8:**
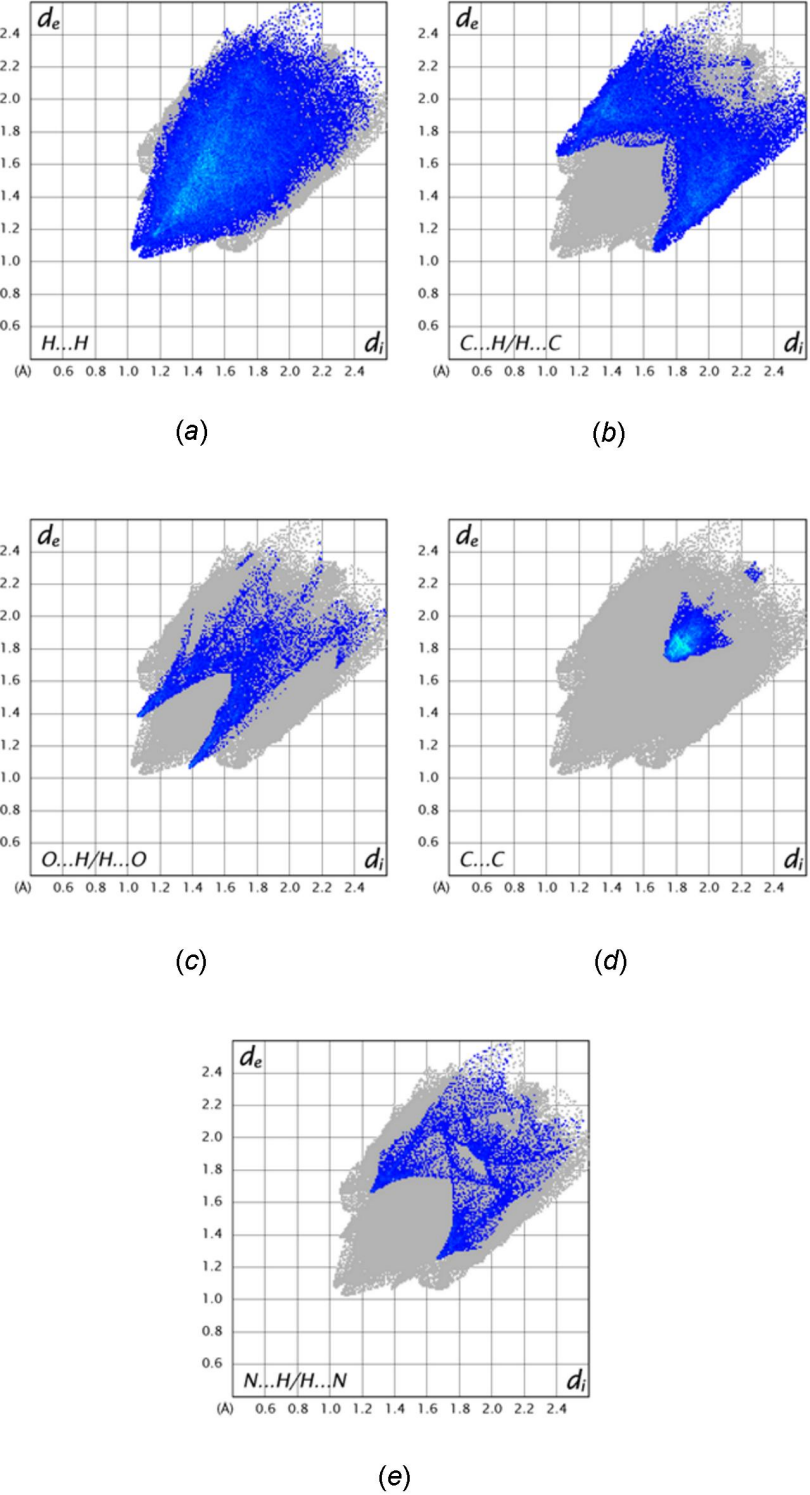
The 2-D fingerprint plots delineated into: (*a*) H⋯H inter­actions, (*b*) C⋯H/H⋯C inter­actions, (*c*) O⋯H/H⋯O inter­actions, (*d*) C⋯C inter­actions and (*e*) N⋯H/H⋯N inter­actions.

To qu­antify the inter­molecular inter­actions, the Hirshfeld surface was calculated with *CrystalExplorer 21.5* (Spackman *et al.*, 2021 ▸). Descriptions of the plots generated and their inter­pretation have been published previously (Tan *et al.*, 2019 ▸). Fig. 6 ▸ shows the *d*_norm_ surface plotted over the range −0.1072 to 1.3548 a.u. together with two neighboring mol­ecules and the connecting C—H⋯O hydrogen bonds. The red spots on the surface clearly indicate the sites of these inter­actions. Fig. 7 ▸ shows the surface plotted over the shape-index with three neighboring mol­ecules included. The pattern of blue and orange triangles marking a site of π-stacking inter­actions is clearly visible in the upper right of the surface with the inter­action denoted by two lines. On the lower left, the C—H⋯π(ring) inter­action is shown by a third line. The 2-D fingerprint plots (Fig. 8 ▸) show that the greatest contribution to the total inter­molecular inter­actions is from H⋯H contacts at 49.6% (Fig. 8 ▸ *a*), which is expected due to the significant hydrogen content and the fact that most of the hydrogen atoms are attached to aromatic rings. The other large contribution is from C⋯H/H⋯C contacts (23.0%, Fig. 8 ▸ *b*), which come primarily from the C—H⋯π(ring) inter­actions. In addition, there are O⋯H/H⋯O contacts (7.4%, Fig. 8 ▸ *c*), C⋯C contacts (5.8%, Fig. 8 ▸ *d*) and N⋯H/H⋯N contacts (5.2%, Fig. 8 ▸ *e*). The C⋯C contacts are primarily the π-stacking inter­actions.

## 6. Synthesis and crystallization

To a solution of 3-phenyl­quinoxalin-2(1*H*)-one (0.5 g, 2.25 mmol) in *N*,*N*-di­methyl­formamide (15 mL) were added 1,3-di­bromo­propane (0.12 ml, 1.125 mmol), sodium hydroxide (0.1 g, 2.25 mmol) and a catalytic qu­antity of tetra-*n*-butyl­ammonium bromide. The reaction mixture was stirred at room temperature for 24 h. The solution was filtered and the solvent removed under reduced pressure. The residue obtained was chromatographed on a silica gel column using a hexa­ne/ethyl acetate 9:1 mixture as eluent. The solid obtained upon solvent removal was recrystallized from ethanol to afford thick, colorless, plate-like crystals of the title compound with a yield of 30%, m.p. = 321–325 K, ^1^H NMR (300 MHz, CDCl_3_) δ ppm: 2.54 (quin, 2H, CH_2_); 3.85 (*t*, 2H, N—CH_2_, *J* = 6Hz); 3.96 (*t*, 2H, O—CH_2_—N, *J* = 6Hz); 7.33–8.12 (*m*, 18H, CHarom).^13^C NMR (75 MHz, CDCl_3_) δ ppm: 22.16 (CH_2_); 33.19 (N—CH_2_); 34.87(N—CH_2_); 113.43–134.23 (CHarom); 134.33–144.11 (Cq); 155.34 (C=O); 155.65 (C=O).

## 7. Refinement

Crystal data, data collection and structure refinement details are summarized in Table 2 ▸. H atoms were positioned geometrically (C—H = 0.93–0.97 Å) and refined as riding with *U*_iso_(H) = 1.2*U*_eq_(H).

## Supplementary Material

Crystal structure: contains datablock(s) global, I. DOI: 10.1107/S2056989024004377/vm2302sup1.cif

Structure factors: contains datablock(s) I. DOI: 10.1107/S2056989024004377/vm2302Isup2.hkl

Supporting information file. DOI: 10.1107/S2056989024004377/vm2302Isup3.cml

CCDC reference: 2354488

Additional supporting information:  crystallographic information; 3D view; checkCIF report

## Figures and Tables

**Table 1:** Hydrogen-bond geometry (Å, °) *Cg*6 is the centroid of the C23–C28 benzene ring.

*D*—H⋯*A*	*D*—H	H⋯*A*	*D*⋯*A*	*D*—H⋯*A*
C3—H3⋯O1^i^	0.93	2.58	3.3871 (16)	146
C10—H10⋯O1	0.93	2.22	2.8531 (16)	124
C27—H27⋯O2^ii^	0.93	2.59	3.4603 (18)	155
C30—H30*A*⋯*Cg*6^iii^	0.97	2.75	3.6013 (14)	147

Symmetry codes: (i) 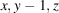 ; (ii) 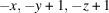 ; (iii) 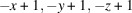 .

**Table 2:** Experimental details

Crystal data	
Chemical formula	C_31_H_24_N_4_O_2_
*M*_r_	484.54
Crystal system, space group	Triclinic, *P* 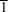
Temperature (K)	298
*a*, *b*, *c* (Å)	9.0384 (3), 9.4484 (4), 14.9524 (6)
α, β, γ (°)	77.267 (1), 83.991 (1), 72.708 (1)
*V* (Å^3^)	1188.16 (8)
*Z*	2
Radiation type	Mo *K*α
μ (mm^−1^)	0.09
Crystal size (mm)	0.35 × 0.30 × 0.10

Data collection	
Diffractometer	Bruker *SMART* *APEX* CCD
Absorption correction	Multi-scan (*SADABS*;Krause *et al.*, 2015 ▸)
*T*_min_, *T*_max_	0.89, 0.99
No. of measured, independent and observed [*I* > 2σ(*I*)] reflections	23105, 6318, 4347
*R*_int_	0.027
(sin θ/λ)_max_ (Å^−1^)	0.686

Refinement	
*R*[*F* ^2^ > 2σ(*F* ^2^)], *wR*(*F* ^2^), *S*	0.046, 0.140, 1.08
No. of reflections	6318
No. of parameters	334
H-atom treatment	H-atom parameters constrained
Δρ_max_, Δρ_min_ (e Å^−3^)	0.30, −0.16

Computer programs: *APEX3* and *SAINT* (Bruker, 2016 ▸), *SHELXT* (Sheldrick, 2015*a* ▸), *SHELXL2019/1* (Sheldrick, 2015*b* ▸), *DIAMOND* (Brandenburg & Putz, 2012 ▸) and *SHELXTL* (Sheldrick, 2008 ▸).

## supplementary crystallographic information

### Crystal data

**Table:**

C_31_H_24_N_4_O_2_	*Z* = 2
*M**_r_* = 484.54	*F*(000) = 508
Triclinic, *P*1	*D*_x_ = 1.354 Mg m^−^^3^
*a* = 9.0384 (3) Å	Mo *K*α radiation, λ = 0.71073 Å
*b* = 9.4484 (4) Å	Cell parameters from 7750 reflections
*c* = 14.9524 (6) Å	θ = 2.4–28.6°
α = 77.267 (1)°	µ = 0.09 mm^−^^1^
β = 83.991 (1)°	*T* = 298 K
γ = 72.708 (1)°	Thick plate, colourless
*V* = 1188.16 (8) Å^3^	0.35 × 0.30 × 0.10 mm

### Data collection

**Table:**

Bruker SMART APEX CCD diffractometer	6318 independent reflections
Radiation source: fine-focus sealed tube	4347 reflections with *I* > 2σ(*I*)
Graphite monochromator	*R*_int_ = 0.027
Detector resolution: 8.3333 pixels mm^-1^	θ_max_ = 29.2°, θ_min_ = 2.3°
φ and ω scans	*h* = −12→12
Absorption correction: multi-scan (*SADABS*;Krause *et al.*, 2015)	*k* = −12→12
*T*_min_ = 0.89, *T*_max_ = 0.99	*l* = −20→19
23105 measured reflections	

### Refinement

**Table:**

Refinement on *F*^2^	Primary atom site location: dual
Least-squares matrix: full	Secondary atom site location: difference Fourier map
*R*[*F*^2^ > 2σ(*F*^2^)] = 0.046	Hydrogen site location: inferred from neighbouring sites
*wR*(*F*^2^) = 0.140	H-atom parameters constrained
*S* = 1.08	*w* = 1/[σ^2^(*F*_o_^2^) + (0.0793*P*)^2^] where *P* = (*F*_o_^2^ + 2*F*_c_^2^)/3
6318 reflections	(Δ/σ)_max_ = 0.001
334 parameters	Δρ_max_ = 0.30 e Å^−^^3^
0 restraints	Δρ_min_ = −0.16 e Å^−^^3^

### Special details

**Table:**

Experimental. The diffraction data were obtained from 3 sets of 400 frames, each of width 0.5° in ω, collected at φ = 0.00, 90.00 and 180.00° and 2 sets of 800 frames, each of width 0.45° in φ, collected at ω = –30.00 and 210.00°. The scan time was 20 sec/frame.
Geometry. All esds (except the esd in the dihedral angle between two l.s. planes) are estimated using the full covariance matrix. The cell esds are taken into account individually in the estimation of esds in distances, angles and torsion angles; correlations between esds in cell parameters are only used when they are defined by crystal symmetry. An approximate (isotropic) treatment of cell esds is used for estimating esds involving l.s. planes.
Refinement. Refinement of F^2^ against ALL reflections. The weighted R-factor wR and goodness of fit S are based on F^2^, conventional R-factors R are based on F, with F set to zero for negative F^2^. The threshold expression of F^2^ > 2sigma(F^2^) is used only for calculating R-factors(gt) etc. and is not relevant to the choice of reflections for refinement. R-factors based on F^2^ are statistically about twice as large as those based on F, and R- factors based on ALL data will be even larger. H-atoms attached to carbon were placed in calculated positions (C—H = 0.95 - 0.99 Å). All were included as riding contributions with isotropic displacement parameters 1.2 - 1.5 times those of the attached atoms.

### Fractional atomic coordinates and isotropic or equivalent isotropic displacement parameters (Å^2^)

**Table:**

	*x*	*y*	*z*	*U*_iso_*/*U*_eq_	
O1	0.39620 (10)	0.88674 (9)	0.06918 (6)	0.0490 (2)	
O2	0.21475 (12)	0.71620 (14)	0.39102 (6)	0.0692 (3)	
N1	0.44699 (10)	0.63291 (10)	0.11355 (6)	0.0352 (2)	
N2	0.21498 (11)	0.64096 (11)	0.00336 (6)	0.0396 (2)	
N3	0.43990 (11)	0.77981 (11)	0.38219 (6)	0.0395 (2)	
N4	0.42341 (11)	0.74089 (11)	0.57324 (7)	0.0408 (2)	
C1	0.28939 (13)	0.50787 (13)	0.06015 (8)	0.0379 (3)	
C2	0.24951 (16)	0.37603 (15)	0.05881 (9)	0.0485 (3)	
H2	0.169960	0.381198	0.022410	0.058*	
C3	0.32574 (17)	0.23990 (15)	0.11018 (10)	0.0546 (4)	
H3	0.299792	0.152567	0.108083	0.066*	
C4	0.44215 (17)	0.23383 (15)	0.16538 (10)	0.0544 (3)	
H4	0.493638	0.141281	0.200596	0.065*	
C5	0.48393 (15)	0.36109 (14)	0.16963 (9)	0.0475 (3)	
H5	0.561308	0.354575	0.207998	0.057*	
C6	0.40876 (13)	0.49995 (12)	0.11567 (7)	0.0362 (3)	
C7	0.36729 (13)	0.77086 (13)	0.06313 (7)	0.0349 (2)	
C8	0.24934 (12)	0.76485 (13)	0.00243 (7)	0.0338 (2)	
C9	0.16777 (12)	0.90221 (13)	−0.06329 (7)	0.0359 (3)	
C10	0.20206 (15)	1.03996 (15)	−0.08165 (9)	0.0502 (3)	
H10	0.280946	1.051677	−0.051211	0.060*	
C11	0.11914 (16)	1.15982 (16)	−0.14513 (10)	0.0563 (4)	
H11	0.142651	1.251697	−0.156303	0.068*	
C12	0.00292 (14)	1.14611 (16)	−0.19197 (9)	0.0492 (3)	
H12	−0.051781	1.227421	−0.234579	0.059*	
C13	−0.03084 (16)	1.01039 (16)	−0.17471 (10)	0.0579 (4)	
H13	−0.108010	0.998776	−0.206648	0.070*	
C14	0.04859 (16)	0.89079 (15)	−0.11040 (10)	0.0529 (3)	
H14	0.021810	0.800486	−0.098289	0.063*	
C15	0.54198 (13)	0.78976 (13)	0.52169 (8)	0.0390 (3)	
C16	0.65404 (14)	0.81762 (15)	0.56774 (9)	0.0480 (3)	
H16	0.645671	0.805913	0.631306	0.058*	
C17	0.77608 (14)	0.86201 (16)	0.52021 (10)	0.0522 (3)	
H17	0.850311	0.880335	0.551234	0.063*	
C18	0.78793 (15)	0.87935 (15)	0.42545 (10)	0.0513 (3)	
H18	0.871213	0.908817	0.393312	0.062*	
C19	0.67912 (14)	0.85389 (14)	0.37802 (9)	0.0465 (3)	
H19	0.688651	0.866859	0.314411	0.056*	
C20	0.55402 (13)	0.80842 (12)	0.42573 (8)	0.0371 (3)	
C21	0.31637 (14)	0.73619 (15)	0.43057 (8)	0.0439 (3)	
C22	0.31797 (13)	0.71537 (13)	0.53167 (8)	0.0387 (3)	
C23	0.19375 (13)	0.66197 (13)	0.58906 (8)	0.0383 (3)	
C24	0.13789 (15)	0.71825 (15)	0.66841 (9)	0.0473 (3)	
H24	0.175355	0.791637	0.683015	0.057*	
C25	0.02711 (16)	0.66568 (17)	0.72557 (10)	0.0569 (4)	
H25	−0.010397	0.704779	0.778051	0.068*	
C26	−0.02799 (16)	0.55617 (16)	0.70545 (10)	0.0543 (3)	
H26	−0.102757	0.521512	0.744151	0.065*	
C27	0.02727 (15)	0.49781 (14)	0.62819 (9)	0.0485 (3)	
H27	−0.008983	0.422527	0.615101	0.058*	
C28	0.13690 (14)	0.55079 (14)	0.56969 (8)	0.0432 (3)	
H28	0.172843	0.511759	0.517023	0.052*	
C29	0.58258 (13)	0.62965 (14)	0.16149 (8)	0.0400 (3)	
H29A	0.619669	0.715413	0.131354	0.048*	
H29B	0.664531	0.538571	0.155101	0.048*	
C30	0.55317 (14)	0.63387 (13)	0.26271 (8)	0.0424 (3)	
H30A	0.652121	0.610476	0.290478	0.051*	
H30B	0.504683	0.555402	0.291637	0.051*	
C31	0.45142 (14)	0.78405 (14)	0.28279 (8)	0.0419 (3)	
H31A	0.495241	0.864656	0.251294	0.050*	
H31B	0.348760	0.804628	0.260318	0.050*	

### Atomic displacement parameters (Å^2^)

**Table:**

	*U*^11^	*U*^22^	*U*^33^	*U*^12^	*U*^13^	*U*^23^
O1	0.0647 (6)	0.0385 (5)	0.0502 (5)	−0.0216 (4)	−0.0196 (4)	−0.0048 (4)
O2	0.0685 (6)	0.1113 (9)	0.0408 (5)	−0.0518 (6)	−0.0143 (4)	−0.0002 (5)
N1	0.0409 (5)	0.0362 (5)	0.0305 (5)	−0.0135 (4)	−0.0033 (4)	−0.0064 (4)
N2	0.0472 (6)	0.0418 (6)	0.0344 (5)	−0.0199 (4)	−0.0024 (4)	−0.0068 (4)
N3	0.0441 (5)	0.0432 (6)	0.0304 (5)	−0.0122 (4)	−0.0047 (4)	−0.0042 (4)
N4	0.0399 (5)	0.0469 (6)	0.0350 (5)	−0.0121 (4)	−0.0046 (4)	−0.0060 (4)
C1	0.0469 (6)	0.0379 (6)	0.0317 (6)	−0.0176 (5)	0.0048 (5)	−0.0081 (5)
C2	0.0617 (8)	0.0467 (7)	0.0462 (7)	−0.0289 (6)	0.0051 (6)	−0.0129 (6)
C3	0.0732 (9)	0.0407 (7)	0.0558 (8)	−0.0274 (7)	0.0155 (7)	−0.0146 (6)
C4	0.0675 (9)	0.0340 (7)	0.0549 (9)	−0.0119 (6)	0.0089 (7)	−0.0037 (6)
C5	0.0539 (7)	0.0389 (7)	0.0451 (7)	−0.0096 (6)	−0.0013 (6)	−0.0043 (6)
C6	0.0431 (6)	0.0357 (6)	0.0307 (6)	−0.0137 (5)	0.0054 (4)	−0.0084 (5)
C7	0.0418 (6)	0.0362 (6)	0.0293 (6)	−0.0148 (5)	−0.0013 (4)	−0.0071 (4)
C8	0.0377 (6)	0.0388 (6)	0.0278 (5)	−0.0148 (5)	0.0008 (4)	−0.0083 (5)
C9	0.0386 (6)	0.0415 (6)	0.0285 (6)	−0.0130 (5)	0.0001 (4)	−0.0072 (5)
C10	0.0562 (8)	0.0510 (8)	0.0483 (7)	−0.0274 (6)	−0.0160 (6)	0.0032 (6)
C11	0.0634 (9)	0.0497 (8)	0.0571 (9)	−0.0278 (7)	−0.0138 (7)	0.0082 (6)
C12	0.0469 (7)	0.0509 (8)	0.0427 (7)	−0.0106 (6)	−0.0065 (5)	0.0028 (6)
C13	0.0578 (8)	0.0569 (9)	0.0609 (9)	−0.0166 (7)	−0.0258 (7)	−0.0042 (7)
C14	0.0584 (8)	0.0439 (7)	0.0602 (9)	−0.0176 (6)	−0.0219 (7)	−0.0052 (6)
C15	0.0366 (6)	0.0405 (6)	0.0378 (6)	−0.0074 (5)	−0.0046 (5)	−0.0067 (5)
C16	0.0451 (7)	0.0571 (8)	0.0416 (7)	−0.0124 (6)	−0.0085 (5)	−0.0096 (6)
C17	0.0408 (7)	0.0564 (8)	0.0619 (9)	−0.0145 (6)	−0.0105 (6)	−0.0122 (7)
C18	0.0433 (7)	0.0488 (8)	0.0613 (9)	−0.0154 (6)	0.0035 (6)	−0.0095 (6)
C19	0.0490 (7)	0.0451 (7)	0.0428 (7)	−0.0126 (6)	0.0025 (5)	−0.0067 (6)
C20	0.0364 (6)	0.0340 (6)	0.0387 (6)	−0.0065 (5)	−0.0038 (5)	−0.0065 (5)
C21	0.0448 (7)	0.0508 (7)	0.0360 (6)	−0.0163 (6)	−0.0080 (5)	−0.0015 (5)
C22	0.0407 (6)	0.0391 (6)	0.0342 (6)	−0.0093 (5)	−0.0048 (5)	−0.0044 (5)
C23	0.0375 (6)	0.0391 (6)	0.0345 (6)	−0.0082 (5)	−0.0063 (4)	−0.0005 (5)
C24	0.0518 (7)	0.0475 (7)	0.0453 (7)	−0.0173 (6)	0.0014 (5)	−0.0118 (6)
C25	0.0626 (9)	0.0630 (9)	0.0471 (8)	−0.0218 (7)	0.0145 (6)	−0.0172 (7)
C26	0.0537 (8)	0.0551 (8)	0.0530 (8)	−0.0226 (6)	0.0049 (6)	−0.0018 (7)
C27	0.0515 (7)	0.0432 (7)	0.0509 (8)	−0.0183 (6)	−0.0108 (6)	0.0011 (6)
C28	0.0474 (7)	0.0441 (7)	0.0369 (6)	−0.0111 (5)	−0.0091 (5)	−0.0045 (5)
C29	0.0394 (6)	0.0434 (7)	0.0382 (6)	−0.0126 (5)	−0.0060 (5)	−0.0069 (5)
C30	0.0516 (7)	0.0405 (7)	0.0337 (6)	−0.0118 (5)	−0.0117 (5)	−0.0019 (5)
C31	0.0501 (7)	0.0429 (7)	0.0303 (6)	−0.0119 (5)	−0.0057 (5)	−0.0021 (5)

### Geometric parameters (Å, º)

**Table:**

O1—C7	1.2222 (13)	C13—H13	0.9300
O2—C21	1.2227 (13)	C14—H14	0.9300
N1—C7	1.3815 (14)	C15—C16	1.3978 (15)
N1—C6	1.3917 (13)	C15—C20	1.4031 (16)
N1—C29	1.4726 (13)	C16—C17	1.3701 (18)
N2—C8	1.2943 (14)	C16—H16	0.9300
N2—C1	1.3787 (15)	C17—C18	1.3864 (19)
N3—C21	1.3830 (15)	C17—H17	0.9300
N3—C20	1.3946 (14)	C18—C19	1.3748 (17)
N3—C31	1.4714 (14)	C18—H18	0.9300
N4—C22	1.2959 (14)	C19—C20	1.3988 (16)
N4—C15	1.3856 (15)	C19—H19	0.9300
C1—C2	1.4006 (15)	C21—C22	1.4819 (16)
C1—C6	1.4039 (16)	C22—C23	1.4818 (16)
C2—C3	1.367 (2)	C23—C28	1.3920 (16)
C2—H2	0.9300	C23—C24	1.3922 (17)
C3—C4	1.384 (2)	C24—C25	1.3818 (17)
C3—H3	0.9300	C24—H24	0.9300
C4—C5	1.3788 (18)	C25—C26	1.3727 (19)
C4—H4	0.9300	C25—H25	0.9300
C5—C6	1.3971 (17)	C26—C27	1.373 (2)
C5—H5	0.9300	C26—H26	0.9300
C7—C8	1.4916 (14)	C27—C28	1.3849 (17)
C8—C9	1.4879 (16)	C27—H27	0.9300
C9—C14	1.3891 (16)	C28—H28	0.9300
C9—C10	1.3892 (16)	C29—C30	1.5162 (16)
C10—C11	1.3840 (18)	C29—H29A	0.9700
C10—H10	0.9300	C29—H29B	0.9700
C11—C12	1.3732 (17)	C30—C31	1.5178 (17)
C11—H11	0.9300	C30—H30A	0.9700
C12—C13	1.3691 (19)	C30—H30B	0.9700
C12—H12	0.9300	C31—H31A	0.9700
C13—C14	1.3785 (18)	C31—H31B	0.9700
			
C7—N1—C6	122.56 (9)	C15—C16—H16	119.7
C7—N1—C29	116.94 (9)	C16—C17—C18	119.41 (12)
C6—N1—C29	120.40 (9)	C16—C17—H17	120.3
C8—N2—C1	120.56 (10)	C18—C17—H17	120.3
C21—N3—C20	122.01 (10)	C19—C18—C17	121.36 (12)
C21—N3—C31	116.59 (9)	C19—C18—H18	119.3
C20—N3—C31	121.26 (10)	C17—C18—H18	119.3
C22—N4—C15	119.21 (10)	C18—C19—C20	119.80 (12)
N2—C1—C2	118.84 (11)	C18—C19—H19	120.1
N2—C1—C6	121.69 (10)	C20—C19—H19	120.1
C2—C1—C6	119.39 (11)	N3—C20—C19	122.91 (11)
C3—C2—C1	120.99 (13)	N3—C20—C15	118.01 (10)
C3—C2—H2	119.5	C19—C20—C15	119.08 (11)
C1—C2—H2	119.5	O2—C21—N3	121.12 (11)
C2—C3—C4	119.07 (12)	O2—C21—C22	123.53 (11)
C2—C3—H3	120.5	N3—C21—C22	115.35 (10)
C4—C3—H3	120.5	N4—C22—C23	117.70 (10)
C5—C4—C3	121.87 (13)	N4—C22—C21	123.37 (11)
C5—C4—H4	119.1	C23—C22—C21	118.92 (10)
C3—C4—H4	119.1	C28—C23—C24	118.47 (11)
C4—C5—C6	119.28 (13)	C28—C23—C22	122.47 (11)
C4—C5—H5	120.4	C24—C23—C22	118.95 (10)
C6—C5—H5	120.4	C25—C24—C23	120.36 (12)
N1—C6—C5	123.06 (11)	C25—C24—H24	119.8
N1—C6—C1	117.58 (10)	C23—C24—H24	119.8
C5—C6—C1	119.37 (11)	C26—C25—C24	120.48 (13)
O1—C7—N1	120.38 (10)	C26—C25—H25	119.8
O1—C7—C8	124.38 (10)	C24—C25—H25	119.8
N1—C7—C8	115.24 (9)	C25—C26—C27	119.99 (12)
N2—C8—C9	117.15 (9)	C25—C26—H26	120.0
N2—C8—C7	122.00 (10)	C27—C26—H26	120.0
C9—C8—C7	120.84 (9)	C26—C27—C28	120.14 (12)
C14—C9—C10	117.63 (11)	C26—C27—H27	119.9
C14—C9—C8	117.09 (11)	C28—C27—H27	119.9
C10—C9—C8	125.28 (10)	C27—C28—C23	120.55 (12)
C11—C10—C9	120.18 (11)	C27—C28—H28	119.7
C11—C10—H10	119.9	C23—C28—H28	119.7
C9—C10—H10	119.9	N1—C29—C30	115.05 (9)
C12—C11—C10	121.47 (12)	N1—C29—H29A	108.5
C12—C11—H11	119.3	C30—C29—H29A	108.5
C10—C11—H11	119.3	N1—C29—H29B	108.5
C13—C12—C11	118.69 (12)	C30—C29—H29B	108.5
C13—C12—H12	120.7	H29A—C29—H29B	107.5
C11—C12—H12	120.7	C29—C30—C31	114.50 (10)
C12—C13—C14	120.56 (12)	C29—C30—H30A	108.6
C12—C13—H13	119.7	C31—C30—H30A	108.6
C14—C13—H13	119.7	C29—C30—H30B	108.6
C13—C14—C9	121.44 (12)	C31—C30—H30B	108.6
C13—C14—H14	119.3	H30A—C30—H30B	107.6
C9—C14—H14	119.3	N3—C31—C30	110.11 (9)
N4—C15—C16	118.36 (11)	N3—C31—H31A	109.6
N4—C15—C20	121.96 (10)	C30—C31—H31A	109.6
C16—C15—C20	119.66 (11)	N3—C31—H31B	109.6
C17—C16—C15	120.69 (12)	C30—C31—H31B	109.6
C17—C16—H16	119.7	H31A—C31—H31B	108.2
			
C8—N2—C1—C2	−179.66 (10)	C20—C15—C16—C17	−0.43 (19)
C8—N2—C1—C6	−2.80 (17)	C15—C16—C17—C18	0.0 (2)
N2—C1—C2—C3	176.47 (11)	C16—C17—C18—C19	0.4 (2)
C6—C1—C2—C3	−0.46 (18)	C17—C18—C19—C20	−0.5 (2)
C1—C2—C3—C4	1.1 (2)	C21—N3—C20—C19	−179.43 (11)
C2—C3—C4—C5	−0.4 (2)	C31—N3—C20—C19	5.04 (17)
C3—C4—C5—C6	−1.1 (2)	C21—N3—C20—C15	0.85 (17)
C7—N1—C6—C5	−177.01 (10)	C31—N3—C20—C15	−174.67 (10)
C29—N1—C6—C5	6.80 (16)	C18—C19—C20—N3	−179.64 (11)
C7—N1—C6—C1	3.35 (15)	C18—C19—C20—C15	0.07 (18)
C29—N1—C6—C1	−172.84 (10)	N4—C15—C20—N3	1.57 (17)
C4—C5—C6—N1	−177.90 (10)	C16—C15—C20—N3	−179.89 (11)
C4—C5—C6—C1	1.74 (18)	N4—C15—C20—C19	−178.15 (10)
N2—C1—C6—N1	1.83 (16)	C16—C15—C20—C19	0.38 (18)
C2—C1—C6—N1	178.67 (10)	C20—N3—C21—O2	177.67 (12)
N2—C1—C6—C5	−177.83 (10)	C31—N3—C21—O2	−6.61 (18)
C2—C1—C6—C5	−0.99 (17)	C20—N3—C21—C22	−2.68 (17)
C6—N1—C7—O1	173.86 (10)	C31—N3—C21—C22	173.04 (10)
C29—N1—C7—O1	−9.82 (16)	C15—N4—C22—C23	179.48 (10)
C6—N1—C7—C8	−6.86 (15)	C15—N4—C22—C21	−0.21 (18)
C29—N1—C7—C8	169.45 (9)	O2—C21—C22—N4	−177.92 (12)
C1—N2—C8—C9	177.83 (9)	N3—C21—C22—N4	2.43 (18)
C1—N2—C8—C7	−1.19 (16)	O2—C21—C22—C23	2.39 (19)
O1—C7—C8—N2	−174.88 (11)	N3—C21—C22—C23	−177.26 (10)
N1—C7—C8—N2	5.88 (16)	N4—C22—C23—C28	−139.78 (12)
O1—C7—C8—C9	6.14 (17)	C21—C22—C23—C28	39.93 (16)
N1—C7—C8—C9	−173.10 (9)	N4—C22—C23—C24	36.31 (16)
N2—C8—C9—C14	7.41 (16)	C21—C22—C23—C24	−143.98 (12)
C7—C8—C9—C14	−173.56 (11)	C28—C23—C24—C25	−0.83 (19)
N2—C8—C9—C10	−172.08 (11)	C22—C23—C24—C25	−177.07 (11)
C7—C8—C9—C10	6.96 (18)	C23—C24—C25—C26	0.7 (2)
C14—C9—C10—C11	−0.2 (2)	C24—C25—C26—C27	0.2 (2)
C8—C9—C10—C11	179.29 (12)	C25—C26—C27—C28	−1.0 (2)
C9—C10—C11—C12	−0.6 (2)	C26—C27—C28—C23	0.87 (19)
C10—C11—C12—C13	0.2 (2)	C24—C23—C28—C27	0.03 (17)
C11—C12—C13—C14	1.1 (2)	C22—C23—C28—C27	176.13 (11)
C12—C13—C14—C9	−2.0 (2)	C7—N1—C29—C30	99.27 (12)
C10—C9—C14—C13	1.5 (2)	C6—N1—C29—C30	−84.34 (12)
C8—C9—C14—C13	−178.05 (13)	N1—C29—C30—C31	−69.98 (13)
C22—N4—C15—C16	179.59 (11)	C21—N3—C31—C30	−92.34 (13)
C22—N4—C15—C20	−1.86 (17)	C20—N3—C31—C30	83.41 (13)
N4—C15—C16—C17	178.15 (12)	C29—C30—C31—N3	−175.74 (9)

### Hydrogen-bond geometry (Å, º)

*Cg*6 is the centroid of the C23–C28 benzene ring.

**Table:**

*D*—H···*A*	*D*—H	H···*A*	*D*···*A*	*D*—H···*A*
C3—H3···O1^i^	0.93	2.58	3.3871 (16)	146
C10—H10···O1	0.93	2.22	2.8531 (16)	124
C27—H27···O2^ii^	0.93	2.59	3.4603 (18)	155
C30—H30*A*···*Cg*6^iii^	0.97	2.75	3.6013 (14)	147

Symmetry codes: (i) *x*, *y*−1, *z*; (ii) −*x*, −*y*+1, −*z*+1; (iii) −*x*+1, −*y*+1, −*z*+1.
